# Aerobic Production of Bacteriochlorophylls in the Filamentous Anoxygenic Photosynthetic Bacterium, *Chloroflexus aurantiacus* in the Light

**DOI:** 10.1264/jsme2.ME20015

**Published:** 2020-05-15

**Authors:** Kazaha Izaki, Shin Haruta

**Affiliations:** 1 Department of Biological Sciences, Tokyo Metropolitan University, 1–1 Minami-Osawa, Hachioji, Tokyo 192–0397, Japan

**Keywords:** *Chloroflexus*, anoxygenic photosynthetic bacteria, bacteriochlorophyll

## Abstract

Filamentous anoxygenic photosynthetic bacteria grow by photosynthesis and aerobic respiration. The present study investigated the effects of light and O_2_ on bacteriochlorophyll contents and the transcription levels of photosynthesis-related genes in *Chloroflexus aurantiacus* J-10-fl ^T^. Under aerobic conditions, *C. aurantiacus* produced marked amounts of bacteriochlorophylls in the presence of light, although their production was strongly suppressed in the dark. The transcription levels of genes related to the synthesis of bacteriochlorophylls, photosystems, and chlorosomes: *bchM*, *bchU*, *pufL*, *pufBA*, and *csmM*, were markedly increased by illumination. These results suggest that *C. aurantiacus* continuously synthesizes ATP by photophosphorylation even in the presence of O_2_.

Filamentous anoxygenic photosynthetic bacteria are a group of anoxygenic photosynthetic bacteria in the phylum *Chloroflexi*, and they grow via photosynthesis and aerobic respiration (Foster *et al.*, 1986; Hanada *et al.*, 1995; Tang *et al.*, 2011; Krzmarzick *et al.*, 2012). Physiological and ecological studies have been conducted on filamentous anoxygenic photosynthetic bacteria, particularly the representative genus *Chloroflexus* (Hanada *et al.*, 1995; Cao *et al.*, 2012). *Chloroflexus* are thermophilic bacteria that are distributed in hot springs (Hanada, 2003; Kubo *et al.*, 2011; Everroad *et al.*, 2012; Otaki *et al.*, 2012; Tank *et al.*, 2017) and are ancient photosynthetic organisms (Blankenship, 1992; 2001). Their ability to fix carbon dioxide by photosynthesis and chemosynthesis has been the focus of intense research on the evolution of autotrophy and its roles in ancient thermophilic ecosystems (Thiel *et al.*, 2014; Hanada, 2016; Nishida *et al.*, 2018; Kanno *et al.*, 2019; Kawai *et al.*, 2019; Martinez *et al.*, 2019). These studies suggested that filamentous anoxygenic photosynthetic bacteria are metabolically versatile and, thus, adaptively alter energy conservation metabolism depending on the surrounding environment and co-existing microorganisms.

The physiological states of photosynthetic organisms are tightly regulated by oxygen (O_2_) tension and light (Pfannschmidt *et al.*, 1999; Bauer *et al.*, 2003) because reactive oxygen species (ROS) generated by the simultaneous presence of O_2_ and light (Elsen *et al.*, 2005; Latifi *et al.*, 2009) are highly toxic for cells. In oxygenic photosynthetic organisms, such as higher plants and cyanobacteria, exposure to strong light has been shown to produce high levels of ROS, which repress photosynthesis activity to avoid the further production of O_2_ (Aro *et al.*, 1993; Nishiyama *et al.*, 2001; 2011). Purple non-sulfur bacteria, which are capable of aerobic respiration as well as anoxygenic photosynthesis, strictly suppress the transcription of photosynthesis-related genes in the presence of O_2_ (Gomelsky and Kaplan, 1995; Ponnampalam *et al.*, 1995; Zeilstra-Ryalls and Kaplan, 1998). In purple non-sulfur bacteria, photosynthesis-related genes are assembled at a limited region in the genome to form a gene cluster and some redox-sensitive transcription factors regulate the transcription of photosynthesis-related genes (Pemberton *et al.*, 1998; Zeilstra-Ryalls and Kaplan, 1998; Igarashi *et al.*, 2001; Bauer *et al.*, 2003). In contrast, photosynthesis-related genes in *Chloroflexus* are located at different regions in genomes (Tang *et al.*, 2011) and the transcriptional responses of genes to O_2_ have not yet been examined. In *Chloroflexus aurantiacus*, a type species of *Chloroflexus*, cellular bacteriochlorophyll (BChl) contents were limited under aerobic dark conditions, but slightly increased with decreases in O_2_ tension in the dark (Foster *et al.*, 1986; Oelze, 1992). However, the effects of O_2_ and light on the synthesis of the photosynthetic apparatus have not yet been systematically elucidated.

In the present study, BChl contents and the transcriptional levels of photosynthesis-related genes in *C. aurantiacus* cells were compared under different cultivation conditions, *i.e.*, aerobic dark conditions, anaerobic light conditions, and aerobic light conditions. The following five photosynthesis-related genes were selected: *bchM* and *bchU* encoding enzymes for BChl synthesis,* pufL* encoding the photosynthetic reaction center L-subunit, *pufBA* encoding the β and α subunits of the B808-866 light-harvesting complex, and *csmM* encoding a chlorosome protein. Chlorosomes, which are uniquely present in filamentous anoxygenic photosynthetic bacteria, are a cellular structural element containing light-harvesting BChls.

*C. aurantiacus* J-10-fl (=DSM 635 ^T^) was obtained from culture collection. The culture was preserved at –80°C using 16% glycerol. The seed culture for the experiment was prepared after reviving the culture of *C. aurantiacus* from the glycerol stock under photoheterotrophic conditions in 1/5 PE medium. 1/5 PE medium contained (L^–1^) 0.1‍ ‍g of yeast extract, 0.1‍ ‍g of casamino acids, 0.1‍ ‍g of sodium acetate, 0.1‍ ‍g of sodium glutamate, 0.1‍ ‍g of sodium succinate, 0.5‍ ‍g of Na_2_S_2_O_3_·5H_2_O, 0.5‍ ‍g of (NH_4_)_2_SO_4_, 0.38‍ ‍g of KH_2_PO_4_, 0.39‍ ‍g of K_2_HPO_4_, 5‍ ‍mL of basal salt solution, and 1‍ ‍mL of a vitamin mixture. The compositions of the basal salt solution and vitamin mixture were described previously (Hanada *et al.*, 1995). Cultivation for analyses was conducted at 55°C using AC medium. AC medium contained (L^–1^) 1.5‍ ‍g of sodium acetate, 0.5‍ ‍g of Na_2_S_2_O_3_·5H_2_O, 0.5‍ ‍g of (NH_4_)_2_SO_4_, 0.38‍ ‍g of KH_2_PO_4_, 0.39‍ ‍g of K_2_HPO_4_, 5‍ ‍mL of basal salt solution, and 1‍ ‍mL of a vitamin mixture (Hanada *et al.*, 1995). pH was adjusted to 7.5 using NaOH.

Anaerobic conditions were achieved by completely filling a screw-capped glass test tube (*ϕ*18‍ ‍mm, 32-mL volume) with medium as reported previously (Pierson *et al.*, 1984). Aerobic cultivation was conducted using 60‍ ‍mL of medium in a 500-mL Sakaguchi flask loosely capped with an aluminum cap by vigorous shaking (200 rpm, BR-40LF; Taitec). Dark conditions were achieved by completely wrapping the cultivation flasks with aluminum foil. A tungsten lamp (4.5‍ ‍μmol m^–2^ s^–1^) was used for light conditions. Precultivation was conducted under anaerobic light and aerobic dark conditions at least once for anaerobic cultivation in the light and aerobic cultivation in the light and dark, respectively. The initial cell density at the inoculation was assessed and adjusted to be 0.005 of optical density (OD) at 610 nm (U-0080D spectrophotometer; Hitachi High-Tech).

A portion of the culture was collected from cultivation vessels and the absorption spectra (600–900 nm) of 50‍ ‍μL were measured with a spectrophotometer (U-0080D; Hitachi High-Tech). Total RNA was extracted from bacterial cells according to Pinto *et al.* (2009). Cells were collected by centrifugation and suspended in 1‍ ‍mL of RNA extraction buffer (PGTX; Pinto *et al.*, 2009). After an incubation at 95°C for 10‍ ‍min, the tubes were placed on ice. One hundred microliters of bromochloropropane was added and mixed well. After incubating at room temperature for 5‍ ‍min, the tubes were centrifuged at 12,000×*g* at 4°C for 15‍ ‍min. The aqueous layer was collected, and nucleic acids were recovered by isopropanol precipitation. The precipitates obtained were dissolved in 30‍ ‍μL RNase free MilliQ water and DNAs were removed using deoxyribonuclease (Mo Bio or Nippon Gene) according to the manufacturer’s instructions. RNAs were purified using the RNeasy Mini Kit (Qiagen). The total RNA concentration was spectrophotometrically assessed using BioSpec-nano (Shimadzu).

cDNA was prepared from extracted RNAs using a reverse transcriptase (ReverTra Ace, Toyobo) and random hexamer as a primer according to the manufacturer’s instructions. Primer sets for the quantitative PCR of genes in *C. aurantiacus* were designed with the help of OligoEvaluator (Sigma-Aldrich, http://www.oligoevaluator.com/OligoCalcServlet) (Table S1). The StepOne Real-time PCR system (Applied Biosystems) was used to quantify DNA fragments with FastStart Universal SYBR Green Master (Roche). The reaction mixture contained 10‍ ‍μL of FastStart SYBR Green Master, 0.2‍ ‍μL of 50 μmol L^–1^ primers, 1‍ ‍μL of cDNA solutions, and 8.6‍ ‍μL of water. Real-time PCR was performed using the following protocol: the first denaturation, 95°C for 10‍ ‍min; denaturation and amplification, 95°C for 15‍ ‍s and 60°C for 60‍ ‍s, respectively (40 cycles). Fluorescence was measured at the end of the amplification step and amplified products were examined by a melting curve analysis from 60 to 95°C. To prepare standard curves for each gene, DNA fragments were amplified using each primer set and the genomic DNA of *C. aurantiacus* by PCR. After the confirmation of specific amplification, PCR products were purified using a PCR purification kit (LaboPass PCR; Cosmo Genetech) and spectrophotometrically quantified with BioSpec-nano (Shimadzu).

BChls were extracted in acetone/methanol (7:2‍ ‍[v/v]) from bacterial cells collected at the exponential phase of growth and absorbance at 767 and 666 nm was measured using a UV-1800 UV-VIS spectrophotometer (Shimadzu). Millimolar extinction coefficients of 76‍ ‍cm^–1^ at 767 nm and 74‍ ‍cm^–1^ at 666 nm were used to assess BChl *a* and BChl *c* contents, respectively (Oelze, 1992). Dry cell weight was measured with harvested cells, washed twice with MilliQ water, and dried at 80°C for 3 d.

*C. aurantiacus* J-10-fl was cultivated in the dark and in the presence of light. No clear differences were observed in growth curves among cultivation conditions (Fig. S1). The absorption spectra of the cultures were compared at the exponential growth phase (Fig. 1). The *in vivo* absorption spectra of cells in anaerobic light showed a peak at 745 nm, corresponding to BChl *c* in cells. Absorbance at 745 nm of cells grown under aerobic dark conditions was quite low, as reported previously (Foster *et al.*, 1986). However, cells cultivated under aerobic light conditions unexpectedly showed a marked absorption peak at 745 nm (Fig. 1).

BChl contents were quantified after extraction from cells grown under each culture condition (Table 1). BChl *c* contents under aerobic light conditions were 17-fold higher than those under aerobic dark conditions and its contents increased further under anaerobic light conditions, as indicated by *in vivo* absorption spectra. BChl *a* contents under anaerobic and aerobic light conditions were also higher than those under aerobic dark conditions. These results suggest that light induced the synthesis of BChl *c* and BChl *a*, and anaerobic conditions further enhanced the synthesis of BChls.

The transcriptional levels of the photosynthesis-related genes, *bchM*, *bchU*, *pufL*, *pufBA*, and *csmM* and the reference gene, *rpoB* were assessed. Fig. 2 shows the transcriptional levels of genes relative to that of *rpoB*. The transcription levels of all the genes tested in cells grown under anaerobic light conditions (black bars) were markedly higher than those under aerobic dark conditions (white bars). Comparisons between light (gray bars) and dark (white bars) conditions under air revealed higher transcription levels of photosynthesis-related genes in the light. Not only genes for BChl synthesis (*bchM* and *bchU*) but also genes for the photosynthetic reaction center (*pufL*) and light-harvesting antenna complex (*pufBA* and *csmM*): *bchM*, *bchU*, *pufL*, *pufBA*, and *csmM* in aerobically illuminated cells showed 25-, 33-, 38-, 9-, and 8-fold higher transcription levels, respectively, than those in cells in the dark. The effects of illumination on the transcription levels of all genes tested under air were significant (*P*<0.05 by the Student’s *t*-test).

In the light, the transcription level of *bchM* under aerobic conditions was similar to that under anaerobic conditions, although the levels of other genes under aerobic conditions were approximately 50% those under anaerobic light conditions (Fig. 2). BchM catalyzes the reaction, Mg-protoporphyrin IX → Mg-protoporphyrin IX monomethyl ester, which is a key step in the biosynthesis of all BChls, including BChl *c* and BChl *a* (Frigaard *et al.*, 2006). The transcription level of *csmM* encoding a chlorosomal protein was higher than those of four other genes in accordance with the large size of this antenna complex (Niedermeier *et al.*, 1994; Orf and Blankenship, 2013).

Based on comparisons of anaerobic light and aerobic dark conditions, filamentous anoxygenic photosynthetic bacteria are considered to suppress the production of the photosynthetic apparatus by responding to O_2_ (Sprague *et al.*, 1981; Foster *et al.*, 1986; Cao *et al.*, 2012). The present study was the first to show BChl production in filamentous anoxygenic photosynthetic bacteria in the presence of O_2_ and substantial transcription levels of all tested photosynthesis-related genes under aerobic conditions in the presence of light. In purple non-sulfur bacteria, the synthesis of the photosynthetic apparatus is strongly suppressed by O_2_ (Ponnampalam *et al.*, 1995; Zeilstra-Ryalls and Kaplan, 1998; Bauer *et al.*, 2003; Elsen *et al.*, 2004), which prevents the production of toxic oxygen species. The present results indicated that filamentous anoxygenic photosynthetic bacteria exhibited completely different environmental responses to those of purple non-sulfur bacteria.

*C. aurantiacus* appears to synthesize ATP by photophosphorylation, even under aerobic conditions; however, illumination did not markedly enhance aerobic growth in *C. aurantiacus*, *i.e.*, doubling times were similar between the two conditions, 10 h, aerobic dark; 12 h, aerobic light (Fig. S1). These results are consistent with previous findings on so-called aerobic anoxygenic photosynthetic bacteria, which produced BChls under aerobic conditions (Shiba *et al.*, 1979; Yurkov and Beatty, 1998a); however, their growth relies completely on aerobic respiration in the presence of light (Yurkov and Beatty, 1998b; Beatty, 2002). As proposed for aerobic anoxygenic photosynthetic bacteria (Imhoff and Hiraishi, 2005), a certain amount of ATP may be provided by cyclic photophosphorylation under aerobic conditions, and the ATP produced may contribute to the maintenance of cell viability when O_2_ and energy sources are depleted. Although aerobic anoxygenic photosynthetic bacteria were shown to reduce BChl contents with illumination (Yurkov and van Gemerden, 1993; Tomasch *et al.*, 2011), *C. aurantiacus* increased the synthesis of the photosynthetic apparatus with illumination, suggesting that it actively utilized the photosynthetic ability under aerobic conditions.

The present study confirmed that the transcriptional levels of all genes tested in *C. aurantiacus* grown in the dark under aerobic conditions were markedly lower than those in the light under anaerobic conditions, but were increased by light irradiation (Fig. 2). These results indicate that *C. aurantiacus* responds to both light and O_2_ in order to regulate the production of the photosynthetic apparatus. BChl *c* in chlorosomes may function as a photoreceptor to regulate the transcription of photosynthesis-related genes because a transcriptional response was observed by illumination with the respective LED light at 450 and 740 nm, which corresponded to the absorption peaks of BChl *c* (data not shown). The results obtained in the present study suggest that the transcription of several photosynthesis-related genes located at several different regions in the genome were simultaneously regulated (Fig. 2). However, transcription factors for photosynthesis-related genes have not yet been identified in filamentous anoxygenic photosynthetic bacteria and genetic modification methods have not been established. In purple non-sulfur bacteria, the transcription factor, PpsR is widely conserved to regulate the expression of a series of photosynthesis-related genes in a gene cluster (Gomelsky and Kaplan, 1995; Pemberton *et al.*, 1998; Oh and Kaplan, 2001; Kovács *et al.*, 2005; Gomelsky *et al.*, 2008). Homologous sequences to genes encoding PpsR were not found in filamentous anoxygenic photosynthetic bacteria, such as *C. aurantiacus* J-10-fl ^T^ (Accession number, NC_010175), *Chloroflexus aggregans* MD-66 ^T^ (NC_011831), *Chloroflexus* *islandicus* isl-2 ^T^ (NZ_LWQS00000000), *Chloroflexus* sp. MS-G (NZ_JPIM00000000), and *Roseiflexus castenholzii* HLO8 ^T^ (NC_009767).

Filamentous anoxygenic photosynthetic bacteria are distributed in densely packed microbial communities as one of the main members with or without cyanobacteria in terrestrial hot springs (Giovannoni *et al.*, 1987; Ley *et al.*, 2006; Klatt *et al.*, 2013). Filamentous anoxygenic photosynthetic bacteria show photoheterotrophy and chemoorganotrophy (Hanada, 2003) as well as photoautotrophy on sulfide or H_2_ as an electron source in some strains (Madigan and Brock, 1975; Holo and Sirevåg, 1986; Strauss *et al.*, 1992; Garrity *et al.*, 2001; Kanno *et al.*, 2019). Kawai *et al.* recently reported that *Chloroflexus* showed H_2_-dependent chemolithotrophy (Kawai *et al.*, 2019). Versatile energy conservation should be effective for survival in environments in which the supply of organic compounds, H_2_, sulfide, and O_2_, may readily fluctuate with the activity of cyanobacteria and flow of sulfidic hot spring water. The photosynthetic apparatus needs to be produced regardless of O_2_ in order to maintain a certain level of cellular ATP under the non-growing state and to quickly initiate photosynthetic growth depending on the availability of electron sources.

## Citation

Izaki, K., and Haruta, S. (2020) Aerobic Production of Bacteriochlorophylls in the Filamentous Anoxygenic Photosynthetic Bacterium, *Chloroflexus aurantiacus* in the Light. *Microbes Environ ***35: **ME20015.

https://doi.org/10.1264/jsme2.ME20015

## Supplementary Material

Supplementary Material

## Figures and Tables

**Fig. 1.**
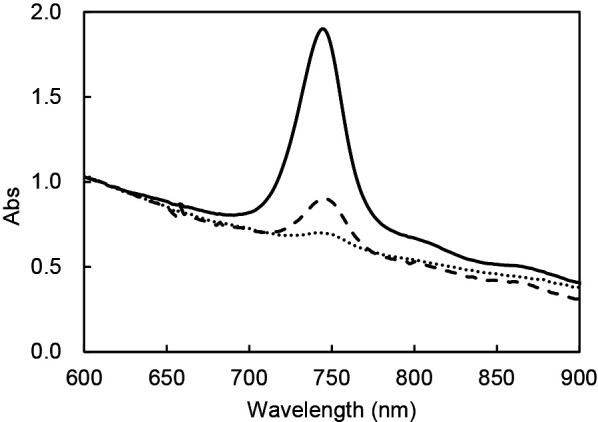
*In vivo* absorption spectra of *Chloroflexus aurantiacus* grown under anaerobic light conditions (solid line), aerobic dark conditions (dotted line), and aerobic light conditions (dashed line) Absorption spectra were measured for cultures at the exponential growth phase. Spectra were adjusted to be Abs at 610 nm=1.0.

**Fig. 2.**
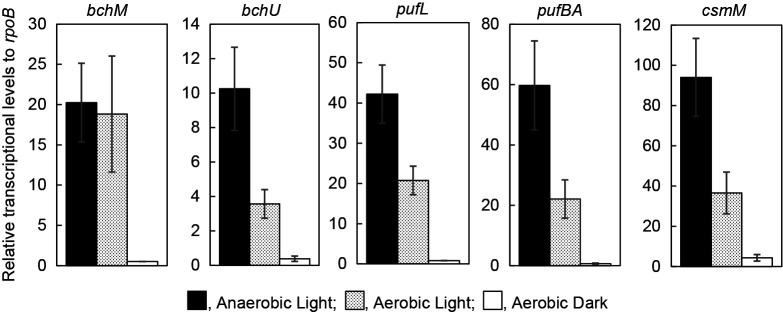
Relative transcriptional levels of *bchM*, *bchU*, *pufL*, *pufBA*, and *csmM* to that of *rpoB* under anaerobic light conditions (black bars), aerobic light conditions (gray bars), and aerobic dark conditions (white bars) mRNA levels were quantified by RT-qPCR to calculate the ratio to that of the housekeeping gene, *rpoB*. All values indicate the average of three independent cultivations. Error bars show the standard deviation of values from three independent cultivations.

**Table 1.** Bacteriochlorophyll contents of *Chloroflexus aurantiacus* cells

Culture conditions	(nmol mg^–1^ of dry cell weight)*	
	BChl *a*	BChl *c*
Anaerobic Light	123.83±8.42	1,285.94±48.42
Aerobic Light	57.71±21.65	332.01±52.46
Aerobic Dark	13.83±21.98	20.01±28.71

*, indicates average values with standard deviations of three independent cultivations.
