# Supplementary material for: Aerobic Production of Bacteriochlorophylls in the Filamentous Anoxygenic Photosynthetic Bacterium, Chloroflexus aurantiacus in the Light
Source: Microbes Environ. 2020 May 15;35(2):ME20015. doi: 10.1264/jsme2.ME20015 (PMC7308566; doi:10.1264/jsme2.ME20015)
Supplement: Supplementary file 1 — Supplementary Material [file 35_20015_s1.pdf]

Supplementary Materials for

Title

Aerobic production of bacteriochlorophylls in the filamentous anoxygenic photosynthetic bacterium, *Chloroflexus aurantiacus* in the light

Authors

Kazaha Izaki and Shin Haruta

Corresponding author

Shin Haruta

Department of Biological Sciences, Tokyo Metropolitan University, 1-1 Minami-Osawa, Hachioji, Tokyo 192-0397, Japan

E-mail: sharuta@tmu.ac.jp; Tel: +81-42-677-2580; Fax: +81-42-677-2559

Table S1. PCR primers designed and used in this study

Target gene	Forward primers (5'-3')	Reverse Primers (5'-3')	Product length
bchM	AACGCTGGTCGGCTATTTAC	GCATCAGACTAAACAGGCCAAC	150 bp
bchU	GGATGCGACAAAAACGAACG	CAGGTCGAACAACCCCAAATC	178 bp
pufL	ACTTTTCGTTCACCGTCGTT	GCCCTACCCAGAAATCAAAG	150 bp
pufBA	CGGCTTTGGTGTCATTGCAG	AAGCAGCGCCACCACAAAAC	166 bp
csmM	TTCGGAGGTGGGATTGTTCG	ATCGCGTGGTAGTGAAGCAA	135 bp
rpoB	ATCTTTGGTGAGAAGGCCCG	TTGTCACCGGCGCTAATCTT	182 bp

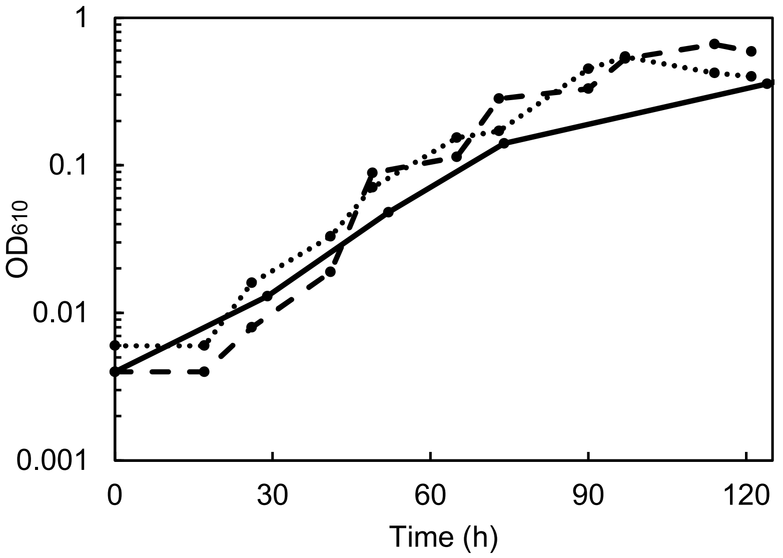

Fig. S1. Growth curve of *C. aurantiacus* J-10-fl grown under anaerobic light conditions (solid line),
aerobic dark conditions (dotted line), and
aerobic light conditions (dashed line)
